# Profiles of subjective well-being among people living with HIV during the COVID-19 pandemic

**DOI:** 10.1007/s11136-023-03493-z

**Published:** 2023-08-10

**Authors:** Marcin Rzeszutek, Ewa Gruszczyńska

**Affiliations:** 1 Faculty of Psychology, University of Warsaw, Stawki 5/7, 00-183 Warsaw, Poland; 2 Faculty of Psychology, SWPS University of Social Sciences and Humanities, Chodakowska 19/31, 03-815 Warsaw, Poland

**Keywords:** HIV, Subjective well-being, Latent profiles, COVID-19

## Abstract

**Background:**

The aim of our study was to examine subjective well-being (SWB) profiles and their sociodemographic and clinical correlates among people living with HIV (PLWH) during the COVID-19 pandemic.

**Methods:**

The participants were 663 PLWH undergoing antiretroviral treatment. Their SWB was evaluated using the Satisfaction with Life Scale and the Positive and Negative Affect Schedule. Sociodemographic and clinical covariates, together with COVID-19 distress, were assessed with a self-report survey.

**Results:**

Latent profile analysis revealed four SWB profiles: average negative, average positive, flourishing and languishing. The languishing profile was the worse in terms of values of SWB components and had a relative overrepresentation of PLWH who were single, without a university degree, and not employed for money. The pandemic-related distress was positively related to being a member of average negative and languishing profiles. Gender and age had no significant effect on either profile membership or directly on the SWB components.

**Conclusion:**

It seems that in the context of chronic illness and socially shared stressful circumstances, which was the COVID-19 pandemic, the components of SWB among PLWH developed rather congruent profiles. Sociodemographic, but not clinical characteristics were found to be significant correlates of belonging to obtained SWB profiles in this sample. The most striking effect with this regards was obtained for the members of the languishing profile, defined by the co-occurrence of low positive affect, low satisfaction with life, and high negative affect.

## Introduction

Despite nearly four decades of intensive empirical research on the tripartite model of subjective well-being (SWB; Diener [1]), there is still no agreement on the internal structure of this model nor on the mutual relationships between its cognitive (life satisfaction) and affective components (positive affect [PA]; negative affect [NA]; see these reviews and meta-analyses: [2–4]). Several studies found that those SWB elements are associated but also constitute independent constructs, especially with respect to their time stability or underlying predictors (e.g., [5, 6]). More specifically, in a meta-analytic review, Busseri [2] observed small to medium associations between SWB indicators and argued for more research on heterogeneous profiles of SWB, which could not be reduced to only high versus low SWB. Relatively recently, Shmotkin [7] created the dynamic and modular model of SWB, which postulates that all the components of SWB can be structured within various individuals in congruous and incongruous ways, and as such, one should explore them simultaneously. However, until now, the dominant methodological attitude to the study of SWB has been a variable‐centered approach, which focuses on the analysis of mean levels of satisfaction with life, PA and NA, and disregards the problem of the heterogeneity of SWB indicators across particular individuals [8, 9]. The latter can be obtained with the aid of the person-centered approach, which has still been rarely used in SWB research (e.g., [10, 11]). In addition, there is a scarcity of studies on the structure of SWB other than in the general population (i.e., a paucity of studies including clinical samples) [2]. In our study, we searched for SWB profiles and their sociodemographic and clinical correlates among people living with HIV (PLWH).

June 2021 marked the 40th anniversary of the first cases of human immunodeficiency virus (HIV) infection detected by the Centers for Disease Control [12], which resulted in a previously unknown illness, acquired immunodeficiency syndrome (AIDS). Since that time, substantial advancement in HIV treatment has transformed HIV from progressing to a terminal condition (AIDS) to now being reclassified as a chronic medical illness [13]. As such, nowadays, the average life expectancy of PLWH does not greatly differ from the life expectancy of healthy individuals in the general population [14]. Nevertheless, PLWH still experience HIV-related distress and consistently declare worse psychological well-being not only compared to the general population but also to patients suffering from other chronic illnesses [15]. Many studies have found that this latter tendency is a derivative of still existing, strong stigmatization of PLWH (see these meta-analyses: [16, 17]). At the same time, several authors have observed that PLWH are also a heterogeneous patient group with respect to coping and adapting to their illness [18–20]. Specifically, although they share the same medical diagnosis (i.e., HIV infection), PLWH display different trajectories in their psychological functioning over time (e.g., [21, 22]). Consequently, applying the person-centered approach to investigating well-being outcomes among PLWH is increasingly recommended [18, 19].

Exploring distinct profiles of SWB of PLWH may be particularly justified during the COVID-19 pandemic as, during this critical period, individual differences in SWB structure may be more pronounced (see the review by [23]. According to a World Health Organization report [24], PLWH were at a 78–95% higher risk of death from COVID-19 in comparison to the general population and also had about a 20% higher risk of hospitalization due to coronavirus infection. In many European countries, access to regular medical care was limited during this period, which translated to a deterioration in adherence to antiretroviral treatment and a significant decline in HIV testing. Additionally, PLWH were under-prioritized for COVID-19 vaccination in approximately 60% of European countries, further exacerbated by misinformation associating COVID-19 vaccines with a risk of HIV infection [25]. All these factors contributed to elevated emotional distress and social isolation within this patient group [23]. On the other hand, studies conducted in the general population showed that the COVID-19 pandemic did not necessarily affect people in a negative way only, that is, some people were either not hampered by the circumstances or even experienced positive changes in their lives (e.g., [26, 27]). The question is whether this heterogeneity of SWB during this pandemic could be observed among PLWH.

### Current study

The aim of our study was to examine SWB profiles and their sociodemographic and clinical correlates among PLWH during the COVID-19 pandemic, including pandemic-related distress. We followed a multivariate approach to SWB and operationalized it via satisfaction with life, PA, and NA. These dimensions were analyzed jointly to identify a group of people characterized by a given profile of SWB instead of analyzing interpersonal differences for each dimension separately. Such an approach has rarely been adopted in existing SWB studies, where components are usually examined individually [2]. Following the person-centered approach, we especially wanted to fill this research gap in the HIV and AIDS literature [19].

There is large literature on the role of sociode- mographic factors (i.e., mostly gender and age) regarding SWB differences in various study populations, but the results are mixed [9]. For example, in large community studies being female was found to be both positively (e.g., [28]) and negatively associated with SWB [29]. The same mixed findings were observed for the role of age, pointing to higher SWB among both younger and older adults [30]. Similar contradictory data on the role of gender and age with regard to SWB can be found in the population of PLWH [15]. Taking into account these results, we examined participants’ gender and age not only as covariates of profile membership, but also checked for their possible main effects on each SWB indicator. In particular, in this study we assumed the presence of effects without specifying them in detail. However, we expected that gender would have an effect on affective SWB components, while age has an effect on satisfaction with life [30–32].

Finally, it was found that SWB is related to various person-dependent and person-independent resources and that some of these associations are more universal, whereas others are probably study- and/or sample-specific [9]. Moreover, some authors have observed the rank-order resistance of SWB to external events in the long term [32], as many of these correlates remain stable or follow predictable trajectories at certain points in life (e.g., health decline with age [6]). In that light, more resourceful personal characteristics in terms of education, employment, intimate relationships, as well as better health status, should be related to more favorable SWB among PLWH as well and, specifically, in the context of the COVID-19 pandemic [23]. Following this line of reasoning, we have formulated three research hypotheses:

#### Hypothesis 1

There will be a heterogeneity of SWB profiles among PWLH observed during the COVID-19 pandemic.

#### Hypothesis 2

In a final profile model, gender and age will have direct effects on the SWB indicators. There will be differences between women and men regarding affective components of SWB. There will also be differences between younger and older PLWH regarding a cognitive component of SWB.

#### Hypothesis 3

Sociodemographic and clinical variables will be correlates of the heterogeneity of SWB, such that PLWH with a more resourceful demographic background and better clinical status will be more likely to be members of a favorable SWB profile (high satisfaction with life: SWL, high positive and low negative affect: PA, NA). Additionally, those who reported higher COVID-19-related distress will be more likely to be members of an unfavorable SWB profile (low SWL, low PA, high NA).

## Method

### Participants and procedure

The participants were 663 adults with medically confirmed diagnoses of HIV infection who were receiving antiretroviral treatment in the specialized outpatient clinic where the study was conducted. The exclusion criteria encompassed being younger than 18 years of age, symptoms of HIV-related cognitive disorders, and current substance abuse, as screened for by medical doctors. The sociodemographic and clinical characteristics of the sample are provided in Table 1.

**Table 1:** Sociodemographic and clinical characteristics of the studied sample (n = 663)

Variable	*n* (%)
Sociodemographic variables	
Gender	
Man	579 (87.3%)
Woman	84 (12.7%)
Age in years (M ± SD)	39.64 ± 9.96
Sexual orientation	
Heterosexual	125 (18.9%)
Other	538 (81.1%)
Relationship	
In a stable relationship	351 (52.9%)
Single	312 (47.95)
Education	
Primary and vocational	56 (8.4%)
Secondary	215 (32.4%)
University degree	392 (59.1)
Employment	
Stable employment	514 (77.5%)
Retirement pension	16 (2.4%)
Ill health pension	60 (9.0%)
Unemployment	73 (11%)
Clinical variables	
HIV and AIDS	
HIV + only	363 (84.9%)
HIV and AIDS	98 (14.8%)
Missing data	2 (0.3%)
HIV infection duration in years (M ± SD)	9.37 ± 8.20
Antiretroviral treatment (ART) duration in years (M ± SD)	7.65 ± 6.48
CD4 count	592.65 ± 243.08

*M* mean, *SD* standard deviation

The study was conducted between July and October 2020 during the so-called “first wave” of the COVID-19 pandemic. After providing informed consent, the participants filled out the study questionnaires in paper or online format, where they could also declare their willingness to take part in further assessments as this study is part of a larger project devoted to the well-being of PLWH. Additionally, they filled out a self-report survey about sociodemographic and clinical information. The study protocol was approved by the local ethics commission.

### Measures

Diener’s tripartite model of subjective well-being (SWB; Diener [1]) was assessed with the Satisfaction with Life Scale (SWLS; [1] along with the Positive and Negative Affect Schedule (PANAS-X). The SWLS comprises five items,respondents assess each item on a 7-point scale ranging from 1 (*strongly disagree*) to 7 (*strongly agree*). Thus, a higher total score on this scale indicates a higher level of satisfaction with life. The Cronbach’s alpha coefficient in the studied sample was 0.88. In the PANAS-X, the participants evaluated the intensity of their affective states during the last month on a 5-point response scale from 1 (*very slightly or not at all*) to 5 (*extremely*). The Cronbach’s alpha coefficients in this study were 0.92 for both subscales.

Distress related to the COVID-19 pandemic was assessed with the question “*How stressful is the COVID-19 pandemic situation for you?*” on a rating scale anchored at 1 (*not at all*) and with an upper value of 5 (*extremely*)*.* Single-item evaluation may be superior to multiple-item rating when the aim is to capture the complete experience of the construct under investigation [33], which is additionally assumed to be unidimensional, clearly defined, and narrow in scope [34].

The sociodemographic and clinical data were collected through a self-report survey due to legal regulations and ethical guidelines concerning sensitive data.

### Data analysis

To analyze the data, we conducted latent profile analysis (LPA [35, 36]). This is a statistical technique used to identify subgroups within a population based on patterns of responses to a set of continuous variables. These patterns are assumed to be explained by a latent categorical variable. This latent variable is thus inferred from the collected empirical data and has a finite number of categories, which correspond to the different latent profiles. It is therefore a person-centered approach as it classifies the participants into subpopulations according to their varying degrees of probability of representing a given pattern of responses. Profiles can differ from each other qualitatively in terms of shape and quantitatively in terms of level, relativized to the sample mean.

When deciding on the selection of the best-fitting profile solution, we followed the steps provided by Ram and Grimm [37]. First of all, we relied on theoretical and content-related considerations. Within this framework, we started by comparing models using relative fit information criteria, namely, the Bayesian information criterion (BIC), the Akaike information criterion (AIC), and the sample size-adjusted Bayesian information criterion (SABIC), for which the lowest values indicate the best-fitted solution [38]. Next, we evaluated the models with respect to the accuracy of the participants’ classification, which is described with an entropy index [39]. Its higher values (i.e., close to 1) indicate a more precise assignment to profiles. Finally, we performed the bootstrap likelihood ratio test (BLRT) to compare the model of interest and a model with one fewer class [40]. Additionally, we took into account the profile size as a small number of cases may suggest lower power, lower precision, and less parsimony [35]. A rule of thumb for the rejection of such profiles is an inclusion of below 1% of the total sample size or fewer than 25 cases [35]. As the data were continuous, we used maximum likelihood (ML) estimation.

After the identification of a final number of profiles, we adopted a bias-adjusted three-step approach to examine the association between the assigned profile membership and covariates [41]. Proportional classification to the profiles was used, along with maximum likelihood bias adjustment [42]. However, as we know from research to date that the key assumption of conditional independence between external variables and latent class indicators may be violated for gender and age, we adopted a correction proposed by Vermunt and Magidson [43], which is also suitable for verifying Hypothesis 2. This can be achieved by including the covariates as predictors not only of the probabilities of latent profile membership but also of the probabilities of response to the indicators within each latent class. Put differently, both gender and age may contribute to the measurement non-invariance of the SWB components across profiles, indicating that the relationship between SWB and its observed indicators differs for men and women, or younger and older PLWH. Therefore, in the model-building strategy, the subsequent step after establishing a profile solution is to examine whether any of the covariates under consideration have a direct effect on the indicators, which can be assessed by examining the bivariate residuals between a covariate and an indicator. In this case, the classification to the profiles should take these effects into account [43].

Additionally, in the third and final step, when testing all the covariates, the classification error adjustment should differ across the values of the covariates that were earlier identified as sources of the measurement non-invariance. Proportional classification to the profiles was used, along with maximum likelihood bias adjustment. All analyses were performed with IBM SPSS Statistics (Version 28.0) and Latent GOLD (Version 6.0.; [44]).

## Results

### Descriptive statistics

Table 2 presents the descriptive statistics for indicators of SWB. On the basis of skewness and kurtosis, it can be said that all variables have a distribution close to normal. The mean value of NA is significantly lower than the mean value of PA (*t* =  − 20.88, *df* = 659, *p* < 0.001), and this effect can be regarded as strong (Cohen’s d =  − 0.81).

**Table 2:** Descriptive statistics for indicators of subjective well-being and COVID-19 distress

Variable	*M*	*SD*	Range	Skewness	Kurtosis
SWL	20.37	6.54	5–35	− 0.28	− 0.51
NA	2.23	0.88	1–5	0.68	− 0.12
PA	3.37	0.81	1–5	− 0.33	− 0.17
COVID-19 distress	2.78	1.15	1–5	0.16	− 0.70

*SWL* satisfaction with life, *NA* negative affect, *PA* positive affect

Due to the cross-sectional character of the data, only a small percentage of missing data was noted, with the highest value equal to 14.5% for the CD4 count. They can be treated as missing completely at random (Little’s MCAR test: χ^2^ = 108.40, *df* = 89, *p* = 0.08). Thus, in further analysis, all available data were used.

### Hypothesis verification

#### Hypothesis 1

**Fig. 1:**
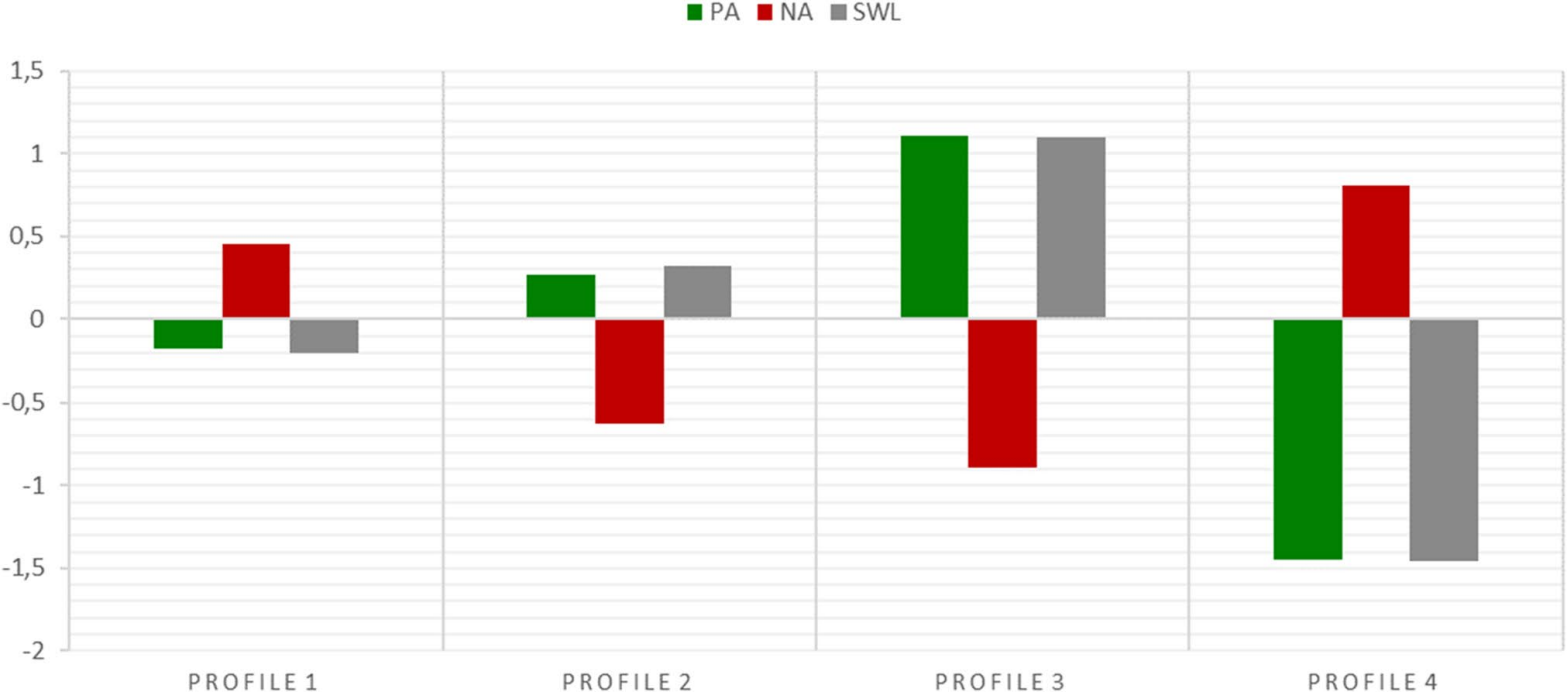
Latent profiles of subjective well-being based on standardized values of positive affect (PA), negative affect (NA) and satisfaction with life (SWL)

Models with one to five profiles were tested. The summary of the results is presented in Table 3. The lowest BIC was obtained for the four-profile solution, whereas the AIC and SABIC continually decreased with the number of profiles, reaching the lowest value for a five-profile model. This decrease, however, was not linear as it diminished with every subsequent profile. Also, the AIC may, in general, suggest more complex models [45]. Moreover, for a five-profile solution, the entropy index is lower than for four-profile solution, and the BLRT, although still significant, yields a smaller difference between four- and five-profile models than between three- and four-profile models. A visual inspection and comparison of the profile plots reveal that in the five-profile solution a profile additional to those obtained in the four-profile solution came from the latest subgroup, which further split into two parallel profiles. Consequently, there was a difference in the intensity of the variables among these profiles, although their shapes remained the same. Therefore, considering all characteristics, the four-profile model was chosen as the most parsimonious and valid representation of the SWB structure in the study sample. This solution is illustrated in Fig. 1.

**Table 3:** Results of latent profile analysis of subjective well-being

Model	AIC	BIC	SABIC	No. of parameters	Entropy	BLRT		Smallest class (modal classification)	
						Value	*p*	% of *N*	Frequency
1 profile	5625	5652	5633	6					
2 profiles	5154	5212	5171	13	0.72	485.47	< 0.001	43.4	288
3 profiles	5071	5161	5097	20	0.68	97.04	< 0.001	19.2	127
4 profiles	5035	5156	5071	27	0.64	49.81	< 0.001	13.3	83
5 profiles	5016	5169	5061	34	0.61	32.55	0.002	14.6	97

*AIC* Akaike Information Criterion, *BIC* Bayesian Information Criterion, *SABIC* Sample Size-Adjusted Bayesian Information Criterion, *BLRT* Bootstrap Likelihood Ratio Test

Profile 1 has the most members (44% of the sample, *n* = 293); it includes PLWH with SWB values close to the sample average and with only a slightly elevated level of NA. This profile will continue to be referred to as *average negative*. Profile 2 (25%, *n* = 163) is characterized by slightly larger deviations from the average and describes participants with generally higher subjective well-being than those in profile 1, which is especially pronounced with regard to NA. This profile will therefore be named *average positive*. Profiles 3 and 4 reflect more strongly the patterns already observed in profiles 2 and 1, respectively. Namely, profile 3 (19%, *n* = 124) consists of *flourishing* participants with PA, SWL above one standard deviation (1.11 and 1.10 *SD*, respectively) from the sample average, and NA 0.90 *SD* below the sample average. In contrast, members of profile 4 (12%, *n* = 83) can be described as *languishing*: they reported NA 0.81 *SD* above the average and PA and SWL 1.45 and 1.46 *SD* below the average, respectively.

#### Hypothesis 2

In the next step, using the four-profile model, we examined whether gender and age, taken separately, had a direct effect on the indicators of SWB. Using bivariate residuals (see Table 4), which are approximate chi-squared statistics with one degree of freedom [42], we found no such effects. Therefore, the classification to the profiles was based on the model without any covariates.

**Table 4:** Testing direct effects of gender and age on the indicators of subjective well-being: bivariate residuals

Covariate	Indicators of subjective well-being		
	SWL	PA	NA
Gender	3.742	2.984	1.121
Age	0.394	1.601	2.176

*SWL* satisfaction with life, *NA* negative affect, *PA* positive affect. A cutoff value based on chi-squared distribution with at *p* = 0.05 with 1 degree of freedom is 3.84

#### Hypothesis 3

Once the profiles were established, their sociodemographic and clinical covariates were examined together with COVID-19-related distress. Table 5 shows the results of the bias-adjusted three-step analysis together with the basic characteristics of the profile members. From 11 analyzed covariates, four were found to be significant: relationship, education, employment, and distress. Pairwise comparisons showed that in profile 4, which describes languishing well-being, there was a relative overrepresentation of PLWH who were single (70%), without a university degree (66%), and not employed for money (45%; i.e., unemployed, in retirement, or on a sickness pension). For COVID-19-related distress, higher values were positively related to the probability of being a member of the average negative profile (*B* = 0.28, *z* = 3.26, *p* = 0.001) and the languishing profile (*B* = 0.31, *z* = 3.65, *p* < 0.001), negatively related to the probability of being a member of the flourishing profile (*B* =  − 0.39, *z* = 3.20, *p* < 0.002), and not significant for the probability of being a member of the average positive profile (*B* =  − 0.19, *z* =  − 1.52, *ns*).

**Table 5:** Basic characteristics of the profile members with results of a bias-adjusted three-step procedure for testing covariates

Covariates	Profile				Wald test
	1 average negative	2 average positive	3 flourishing	4 languishing	
	n = 293	n = 163	n = 124	n = 83	
Sociodemographic variables					
Gender (man)	91%	85%	90%	75%	2.39
Age (M ± SD)	40.2 ± 10.0	40.5 ± 10.2	39.1 ± 9.0	38.6 ± 9.8	4.00
Sexual orientation (other than heterosexual)	82%	81%	86%	71%	2.03
Relationship (single)	45%	44%	40%	70%	8.22*
Education (university degree)	57%	70%	68%	34%	15.69*
Employment (stable)	77%	82%	87%	55%	19.48*
Clinical variables					
HIV infection duration (M ± SD)	9.5 ± 8.0	10.4 ± 8.4	10.0 ± 8.7	8.8 ± 8.2	2.02
ART duration (M ± SD)	7.7 ± 6.4	8.6 ± 7.1	7.7 ± 6.8	6.8 ± 6.6	1.81
CD4 count (M ± SD)	575.0 ± 247.4	602.1 ± 235.8	623.2 ± 242.4	564.0 ± 213.5	0.77
AIDS diagnosis	15%	16%	11%	18%	2.63
COVID-19 distress (M ± SD)	3.0 ± 1.2	2.5 ± 0.9	2.4 ± 1.1	3.0 ± 1.2	32.22**

*M* mean, *SD* standard deviation

*p < 0.05, **p < 0.001

## Discussion

The results of our study are in accordance with our first research hypothesis, as we observed heterogeneity of the SWB profiles among PWLH. However, the pattern of the profiles obtained was intriguing. Namely, the largest and second-largest groups of participants belonged to, respectively, the average negative profile (profile 1), with SWB values close to the sample average, and the average positive profile (profile 2), with slightly larger deviations from the average and generally higher SWB. The next two profiles more strongly reflected the patterns already observed in the average profiles. Specifically, profile 3 comprised flourishing participants with PA and SWL above one standard deviation from the mean, and profile 4 comprised languishing participants, with high NA and very low PA and SWL. On the one hand, the profiles obtained provide support for those SWB theories that highlight the bidirectional association between cognitive and affective aspects of well-being such that life satisfaction enhances positive affect or vice versa, but they are both oppositely linked to NA [4]. On the other hand, however, the most highly contrasting profiles comprise less than 25% of the sample, revealing that the typical profiles for PLWH in our study are rather flat, i.e., average negative and average positive, with small differences making them better or worse in terms of SWB. Flourishing and, more importantly, languishing can therefore be considered as less frequent characteristics than simply slightly better or slightly worse SWB. This is a significant finding in the context of this study.

Trying to interpret aforementioned result it should be stated that satisfaction with life concerns the global evaluation of life and, thus, is more driven by external events, whereas affective well-being is grounded more on the evaluation of recent activities and, therefore, can be much more transient and dynamic over time. For example, Luhmann et al. [6], in a meta-analytic review, found that various critical life events (e.g., divorce, job loss, retirement) may leave their footprint much more indelibly on cognitive components of SWB than on affective ones. In contrast, affective components of SWB are much more fluid and originate mostly in personality traits [46]. The COVID-19 pandemic constituted for PLWH a critical life event, resulting in the previously mentioned substantial disruption in their medical care and social life [23]. However, the event, by virtue of its scale and duration, also initiated a socially shared chronic stress. It was therefore interesting that within the same clinical sample in the similar context of uncontrollable external circumstances, we observed two such contrasting profiles, i.e., *flourishing* versus *languishing* [47]*,* which is another argument for PLWH being a heterogeneous patient group with regard to adaptation not only to HIV infection but also in terms of general functioning [19, 20].

Our study also provided some support for our third research hypothesis but yielded a null result with regard to the second hypothesis. Specifically, as expected, intimate relationship status, education level, and current employment status were significant correlates of the probability of membership of SWB profiles, which supports the third hypothesis. Additionally, COVID-19-related distress was positively related to membership in the less-favorable profiles of SWB. To some extent, these findings can be drawn intuitively and are in line with previous studies on PLWH, as well as with studies on psychological functioning during the COVID-19 pandemic (e.g., [23]. However, in contrast to our second hypothesis, there was no effect of gender and age not only in terms of profile membership but also in terms of the SWB components in these profiles. These results may contribute valuable insights to the existing literature on the psychological well-being of PLWH [15]. It is noteworthy that the majority of well-being studies in this population have predominantly employed a variable-focused approach, neglecting to consider the potential influence of various sociodemographic factors, which often extend beyond individuals' personal control, on the heterogeneity of SWB profiles among PLWH [48]. This oversight bears significant implications, both in theoretical and practical terms, for the development of effective interventions targeting individuals and specific groups [49], particularly given the growing disparity observed between advancements in disease management and the mental health status of PLWH [50].

Interestingly, in line with the above, it is worth noticing that clinical variables describing the duration of the disease and its current state (i.e., years since diagnosis, years of ART, AIDS diagnosis and CD4 count) in our study had no effect on profile membership. This finding might be specific to our sample (which we will discuss in more detail in the limitations section). However, it highlights the importance of acknowledging resources beyond those directly linked to the disease, particularly in the context of coping with a chronic somatic condition. These resources are often overlooked in medically oriented interviews but may play a crucial role in functioning, especially when somatic symptoms can be well managed through good compliance but remain incurable, resulting in ongoing psychological and social implications [51]. In this sense, the findings support the basic premise of the conservation of resources theory [52], which, although not an SWB theory, highlights the role of broadly understood resources in human functioning, especially in the face of stress, with a social perspective that goes beyond exclusively individualized coping behaviors [53]. For instance, as Das et al. [9] showed in a systematic review, not only socioeconomic status (i.e., income, education, employment, family structure, and immigration status) but also religion, culture, and geographical location may be relevant for individual SWB. Similar ideas have been discussed in light of the effect of cross-country differences on SWB-related indexes [54, 55]. Consequently, this gap neglecting the systematic inclusion of more structural and basic factors together with a variable-oriented approach contributes to contradictory results and an incomplete picture of SWB determinants and correlates in the literature [9, 19, 56, 57]. The novelty of our study is, thus, our analysis of the heterogeneity of PLWH with regard to their sociodemographic and clinical characteristics and associations with SWB profiles, particularly in the critical time of the pandemic.

Finally, we obtained results in line with the growing body of research that applies a person-centered approach to SWB [10, 11, 58]). These findings showed that mixed SWB profiles (i.e., those with incongruent values for each component) can differently impact various areas of psychological functioning. Accordingly, disregarding the mutual interplay between well-being indicators and solely adhering to unidimensional relationships, as advocated by the variable-centered approach, hinders a comprehensive understanding of well-being structure and dynamics. It appears that SWB operates through various modules, engaging in a transactional process influenced by both internal and external factors [7]. Moreover, the SWB components demonstrate varying stability levels over the life course. This observation aligns with our own research findings, which suggest that profiles likely strive for internal consistency. Hence, alongside the height of the profiles, this internal consistency (or lack thereof) emerges as another crucial characteristic. To these characteristics should also be added a susceptibility to change and the persistence of these changes, which is also subject to marked individual differences [59].

Consequently, to ensure further progress in this research area, SWB should be described in a way that takes into account all these parameters and extends beyond testing the intensity of isolated components. This broader conceptualization of SWB may be particularly useful in the case of clinical samples similar to PLWH, whose well-being has constantly been challenged due to chronic HIV-related stress and still-existing strong social stigmatization [15–17], which was further strengthened during the COVID-19 pandemic [23].

### Strengths and limitations

This study has several strengths, such as the large clinical sample of PLWH examined during the unique social context of the common health threat due to the COVID-19 pandemic. However, there are also some limitations to this research. First, the cross-sectional design makes it impossible to draw any cause-and-effect conclusions. Secondly, our sample, yet large, cannot be treated as representative for PLWH. Specifically, our participants were highly functioning with relatively good control of their HIV infection and—considering the mean levels of their affective components of SWB—with higher PA than NA. Third, we used a single item to measure COVID-related stress and we did not mention other than distress factors of individual experiences of the COVID-19 pandemic, including obstacles to adherence to treatment, consequences of changes in medical care, and differences in social attitudes toward PLWH. Finally, regarding ethical and legal issues associated with data protection, with the exception of medically confirmed diagnoses of HIV infection, other clinical variables were self-reported.

## Conclusion

This study contributed to the literature on SWB among PLWH by applying a person-centered approach that provides data beyond and complementary to what can be gleaned by a variable-centered approach only. More specifically, it seems that SWB is not the sum of the tripartite components of Diener’s model [1, 3] but rather a complex and dynamic system. In addition, the components of SWB develop congruent profiles, also in the context of chronic illness and socially shared stressful circumstances. Additionally, their extreme intensity, in both directions (flourishing *versus* languishing), is observed in less than 25% of the sample. PLWH with fewer personal and socially valid resources belonged to the worst SWB profile, which was independent of their clinical characteristics. This picture of SWB is particularly informative given the chronic nature of medical and social stressors among PLWH [15–17] and the unique context of this study.

## Data Availability

All the data are available upon the request from the corresponding author.
